# Supplementary material for: Decoding the JAK-STAT Axis in Colorectal Cancer with AI-HOPE-JAK-STAT: A Conversational Artificial Intelligence Approach to Clinical–Genomic Integration
Source: Cancers (Basel). 2025 Jul 17;17(14):2376. doi: 10.3390/cancers17142376 (PMC12293912; doi:10.3390/cancers17142376)
Supplement: Supplementary file 1 [file cancers-17-02376-s001.zip › cancers-3718749-supplementary.pdf]

Supplementary Materials:

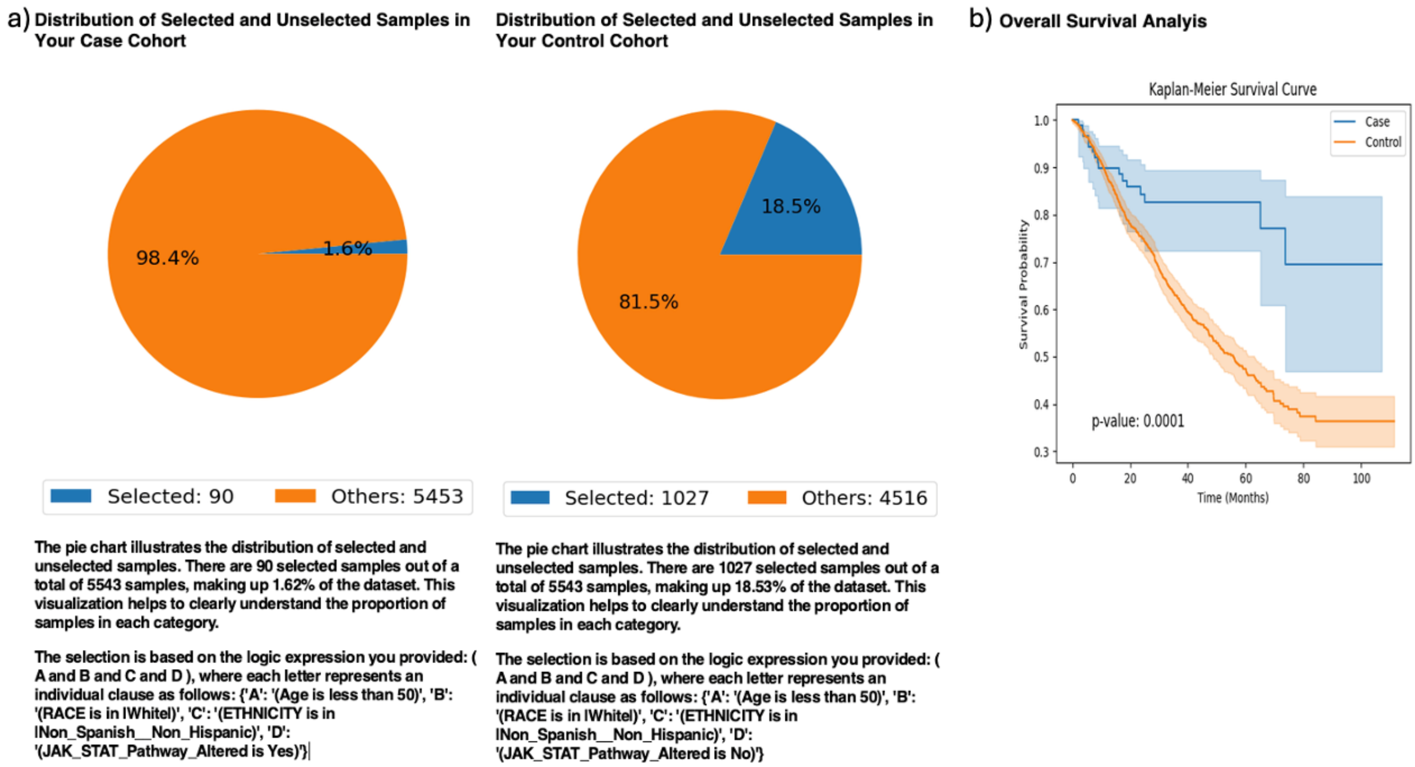

Figure S1. AI-HOPE-JAK-STAT analysis of early-onset colorectal cancer in patients stratified by JAK/STAT pathway alteration status. This figure presents the outcome of a natural language-driven query performed using AI-HOPE-JAK-STAT to evaluate the association between JAK/STAT pathway alterations and overall survival in early-onset colorectal cancer (EOCRC) patients of Non-Hispanic White (NHW) background. a) Pie charts display the proportion of selected versus unselected samples within each cohort relative to the full colorectal cancer dataset (n = 5543). The representation highlights the relative infrequency of JAK/STAT pathway mutations in this subgroup, yet sufficient for comparative survival analysis. b) The Kaplan-Meier survival plot demonstrates a marked and statistically significant difference in survival outcomes between the two groups. Patients with JAK/STAT alterations (blue) exhibited considerably improved survival compared to their non-altered counterparts (orange). The p-value of 0.0001 from the log-rank test confirms the robustness of this association. The narrow confidence intervals further support the reliability of the observed survival benefit, suggesting a potentially protective or biologically meaningful role of JAK/STAT pathway alterations in EOCRC among NHW individuals.

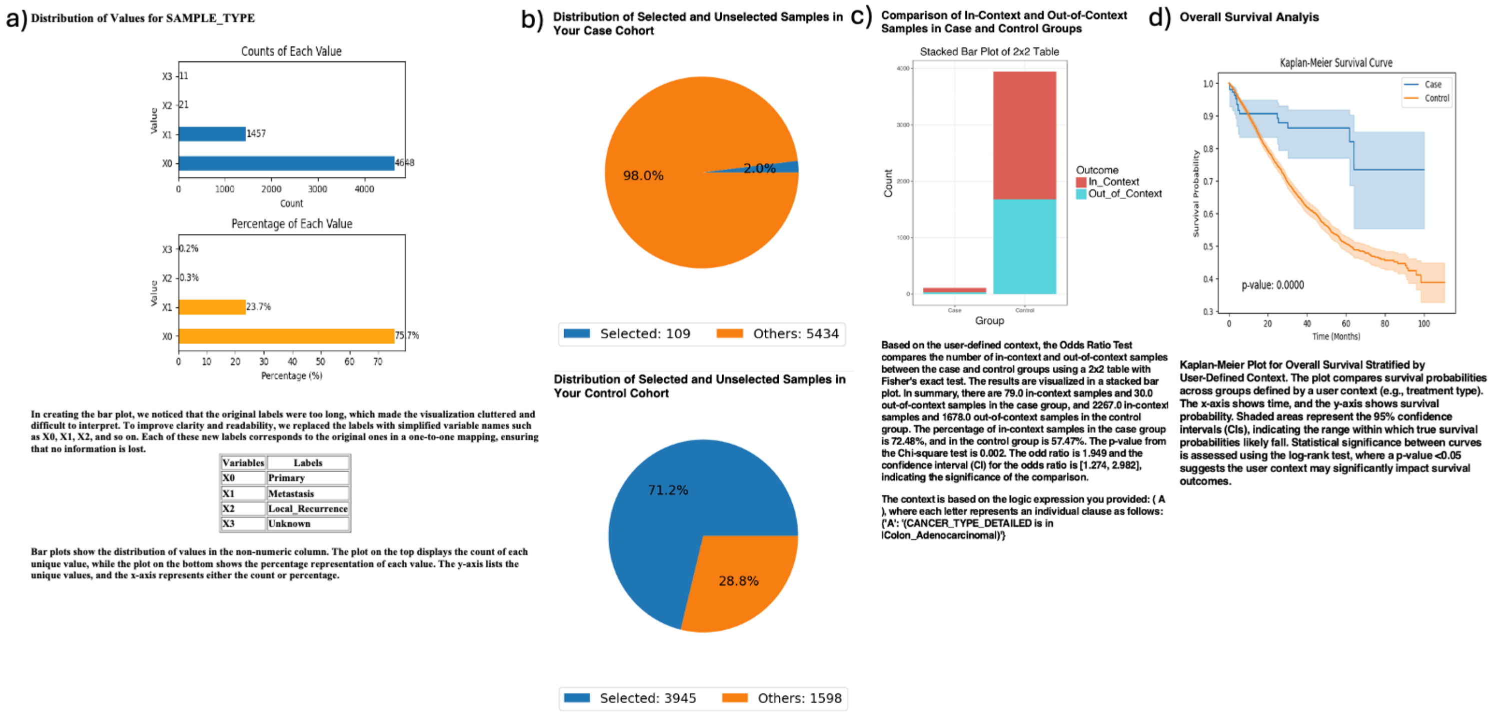

Figure S2. AI-HOPE-JAK-STAT analysis of STAT5B-mutant primary colorectal tumors with odds ratio context in colon adenocarcinoma. This figure highlights the application of AI-HOPE-JAK-STAT to assess survival outcomes and mutation enrichment among colorectal cancer (CRC) patients with STAT5B mutations in primary tumor samples. The case cohort includes primary tumors harboring STAT5B mutations (n = 109), while the control cohort includes primary tumors without such mutations (n = 3,945). a) Bar plots summarize the distribution of sample types across the dataset using simplified labels (e.g., X0 = Primary, X1 = Metastasis), confirming that the majority of samples are primary tumors. b) Pie charts illustrate the selection of case and control cohorts based on mutation status. STAT5B-mutated primary tumors represent 2.0% of the dataset, while STAT5B wild-type cases account for 71.2%, indicating sufficient data availability for comparative analysis. c) An odds ratio test evaluates the enrichment of colon adenocarcinoma cases across the defined cohorts. A 2×2 table compares in-context (colon adenocarcinoma) and out-of-context samples in both case and control groups. Among the case group, 72.48% were in-context, compared to 57.47% in the control group. The resulting odds ratio is 1.949 (95% CI: 1.274–2.982; p = 0.002), suggesting a statistically significant association between STAT5B mutations and colon tumor location. d) Kaplan-Meier survival analysis demonstrates significantly better overall survival in patients with STAT5B mutations (blue) compared to those without (orange), with a p-value of < 0.0001. The clear separation of survival curves and narrow confidence intervals support a potential prognostic benefit of STAT5B mutation status in CRC patients with primary tumors.

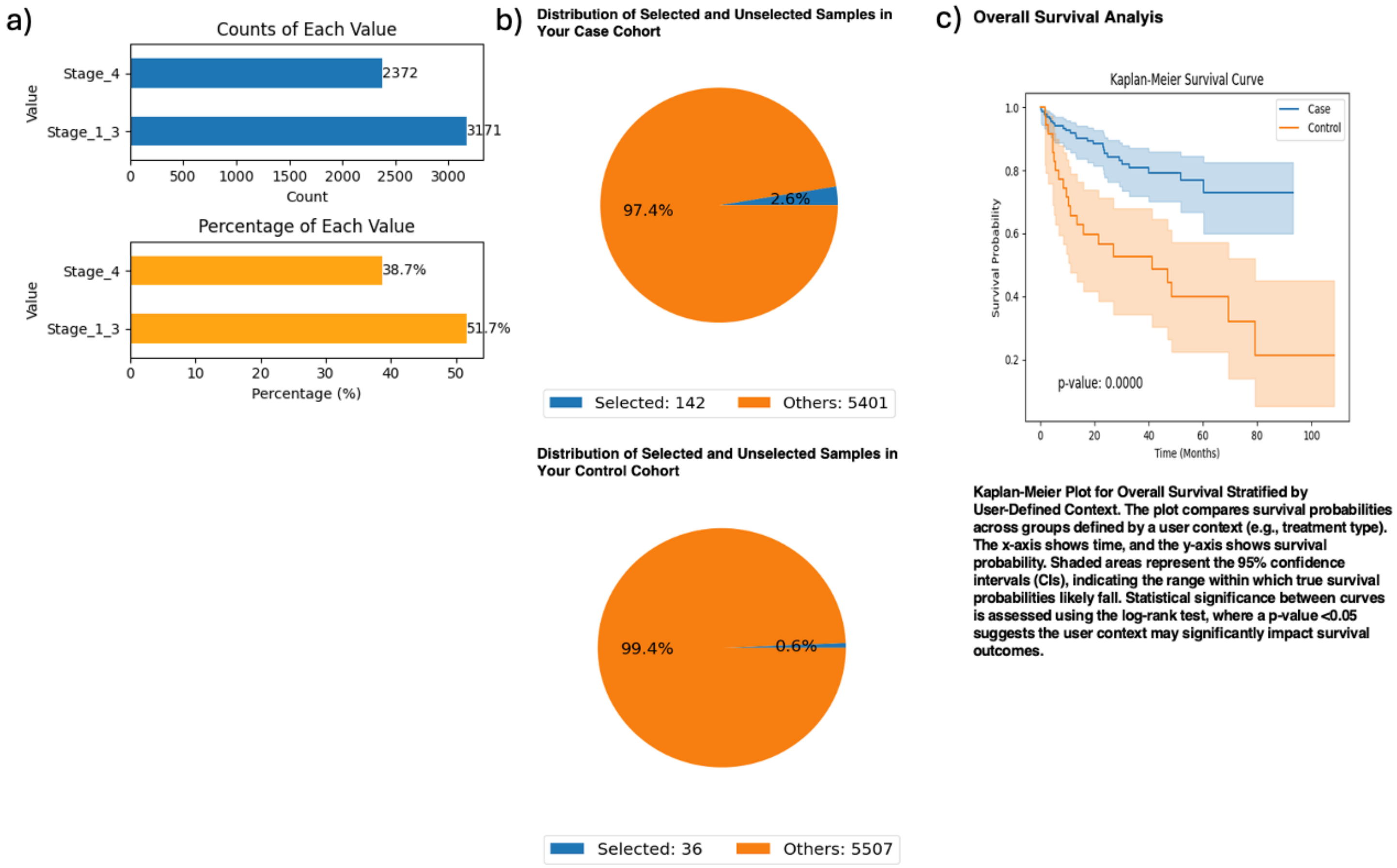

Figure S3. AI-HOPE-JAK-STAT analysis of colorectal cancer patients with JAK3 mutations stratified by tumor stage. This figure demonstrates how AI-HOPE-JAK-STAT enables stratified survival analysis in colorectal cancer (CRC) patients carrying JAK3 mutations, comparing those with early-stage (Stage I–III) versus advanced-stage (Stage IV) disease. a) Bar plots show the distribution of patients across tumor stages, with 51.7% in Stage I–III (n = 3,171) and 38.7% in Stage IV (n = 2,372). These distributions confirm the presence of adequate sample sizes across stage groups to support comparative analyses. b) Pie charts display the relative proportions of selected JAK3-mutant patients in each cohort. The early-stage group includes 142 samples (2.6% of the total dataset), while the advanced-stage group includes 36 samples (0.6%). This visualization reflects the lower prevalence of JAK3 mutations among late-stage CRC cases. c) Kaplan-Meier survival analysis reveals a highly significant difference in overall survival between the two groups ($p < 0.00001$). Patients with JAK3 mutations diagnosed at Stage I–III (blue) demonstrate markedly improved survival relative to those diagnosed at Stage IV (orange). The wide separation between survival curves and narrow confidence intervals reinforce the strength of the association, suggesting that tumor stage remains a strong prognostic factor even among JAK3-mutated CRC patients.
